# Therapeutic Effect of Liquiritin Carbomer Gel on Topical Glucocorticoid-Induced Skin Inflammation in Mice

**DOI:** 10.3390/pharmaceutics16081001

**Published:** 2024-07-28

**Authors:** Yun Zhang, Sijia Li, Yanfang Huang, Congjing Song, Weiqiang Chen, Yiling Yang

**Affiliations:** 1 School of Nursing, Guangdong Pharmaceutical University, Guangzhou 510006, China; 2 School of Basic Medical Sciences, Guangdong Pharmaceutical University, Guangzhou 510006, China

**Keywords:** liquiritin, glucocorticoid, skin barrier, inflammation

## Abstract

Glucocorticoids are often used and highly effective anti-inflammatory medications, but prolonged topical application may alter the epidermis’ normal structure and function, potentially resulting in a number of adverse effects. Topical glucocorticoid-induced skin inflammation is a dangerous condition that develops after topical glucocorticoid use. The patients become dependent on the medication and, even after the medication is stopped, the dermatitis symptoms recur, severely impairing their quality of life. Thus, the need to aggressively confront Topical glucocorticoid-induced skin inflammation is critical. Prior research has demonstrated that topical administration of licorice’s flavonoid component liquiritin stimulates epidermal proliferation, which in turn enhances the creation of collagen and the healing of wounds. Therefore, the purpose of this work was to determine if topical use of liquiritin carbomer gel can treat glucocorticoid-induced changes in mice skin epidermal function, and the mechanisms involved. The findings demonstrated that, in the mice model of topical glucocorticoid-induced skin inflammation, liquiritin carbomer gel aided in the restoration of skin barrier function. These outcomes may have been caused by enhanced expression of the proteins Aquaporin 3, Keratin 10, and Claudin-1, as well as the restoration of epidermal hyaluronan content. In the meantime, liquiritin carbomer gel dramatically decreased the expression of TNF-α, IL-1β, IL-6, IFN-γ, and IgE in mice, according to ELISA tests. Furthermore, topical treatment of liquiritin carbomer gel boosted the expression of superoxide dismutase, catalase, and decreased malondialdehyde expression, potentially counteracting the detrimental effects of glucocorticoids on the epidermis. In summary, these findings imply that topical liquiritin carbomer gel can treat glucocorticoid-induced skin damage through various mechanisms of action.

## 1. Introduction

Glucocorticoids (GC) are potent endogenous compounds that act through a variety of mechanisms to modulate the immune system, attenuate inflammation, and influence cell proliferation. In clinical practice, GC is frequently used to treat a variety of inflammatory skin conditions, such as psoriasis, eczema, and atopic dermatitis. However, a number of adverse effects, including skin atrophy [1], capillary dilatation, and topical glucocorticoid-induced skin inflammation (TGSI), can arise from topical GC drug use. TGSI is an inflammatory skin disease that develops when topical GC is used improperly over an extended period of time and results in skin damage after the medicine is stopped. TGSI is characterized by recurring face erythema, papules, peeling, telangiectasia, and rashes that resemble both acne and rosacea, while the exact pathophysiology of the condition is still unknown. Symptoms, like skin tightness, stinging, burning, and dryness, are common and have a negative impact on patients’ emotional and physical well-being [2,3]. Prolonged pain and suffering can result in social difficulties, anxiety, and depression, which can negatively impact the patient’s psychological health and quality of life [4]. Nowadays, a large range of GC is readily available for purchase at pharmacies in China. Patients frequently purchase and utilize GC against medical advice and independently, since doctors do not provide adequate information in this area. In an attempt to achieve curative benefits, some people even go so far as to increase the dosage without authorization, which can quickly result in GC misuse. Furthermore, in order to make money or to stay in the market, some private practices and beauty associations add GC ingredients to their goods without permission. In order to promote the benefits of meeting client needs, they also employ whitening, spot removal, and other gimmicks. The prevalence of GC skin illnesses has increased in China as a result of these variables.

Since the pathogenic mechanism of TGSI is yet unknown, several investigations have shown a strong correlation between the condition’s incidence and damage to the skin barrier [5,6]. Antihistamine, anti-inflammatory, hormone-reducing, and replacement therapy are the main treatments for TGSI. Hormone replacement therapy is currently the most often utilized form of treatment [7]. This treatment works well to relieve burning and itching, but it is ineffective at improving facial flushing, erythema, and dilated capillaries. Furthermore, the illness frequently returns and worsens over time, putting the sufferer under a great deal of mental strain. People’s standards of social existence are rising and their ideas of aesthetics are evolving in the modern world. For this reason, finding natural herbs that are effective and have minimal toxicity and adverse effects is crucial to both preventing and treating this illness [8].

The dried root and rhizome of Glycyrrhiza glabra, Glycyrrhiza uralensis Fisch, or Glycyrrhiza inflata Bat, is known as licorice, and it is utilized extensively as a natural sweetener and commercial and medical herb [9,10]. One of the primary components of licorice’s flavonoids is liquiritin (LQ). It has demonstrated a variety of pharmacological properties, including cardiovascular protection [11], antioxidant activity [12], and anti-inflammatory properties [13]. In the area of treating skin conditions, Li et al. demonstrated that LQ could increase the synthesis of collagen, which served as a barrier to protect the skin, while also reducing the release of proinflammatory factors and ROS in the UVB-induced skin of SD rats [14]. Additionally, the earlier research showed that LQ enhanced wound healing and repair in mice with solar dermatitis and decreased the production of inflammatory factors in HaCat cells when exposed to UVB [15].

Though LQ has been shown to improve skin health, its effectiveness and function in TGSI are still unclear. Therefore, this study examined the topical administration of LQ in order to determine its potential for mitigating the symptoms of TGSI and to verify its therapeutic role.

## 2. Materials and Methods

### 2.1. LQ–CG Preparation

Carbomer 940 (Meilunbio, Dalian, China) is a gelling agent that is utilized as a drug carrier for liquiritin carbomer gel (LQ–CG), since it has superior transdermal absorption qualities and is free of stimulants. LQ was acquired from Chengdu Nakeli Biotechnology Co., Ltd. (Chengdu, China) with a purity of >98% and a CAS of 551-15-5. The Supplementary Materials contain the structural formula for LQ (Figure S1). The three various LQ concentrations were combined, the mixture was agitated for five minutes, and then triethanolamine (Macklin, Shanghai, China) was added to adjust the pH to 7.0. The combination continued to swirl at 25 °C until a uniform, excellent gelatinous material was formed. Specifically, it was effectively possible to create LQ–CG with 0.5%, 1%, and 2% (*w*/*v*) [16].

### 2.2. Establishment and Treatment of a Mice Model of TGSI

Before the experiment, approval was obtained from the Animal Ethics Committee (No. gdpulacspf2022471), and the animals were handled in compliance with the experimental protocols outlined in the Guangdong Pharmaceutical University Guidelines for the Care and Use of Laboratory Animals. The day before modeling, 56 BALB/C mice were depilated on the back with a nonirritating depilatory cream following a week of acclimation feeding. The skin was exposed and measured at approximately 2 cm × 2 cm (length and width). Except for the normal control (NC) group, the remaining mice received topical applications of 0.05% clobetasol propionate cream (Guilin, China) on their backs, administered twice daily for 21 consecutive days. Application of the medicine was discontinued on day 22 of the experiment. After the mice’s skin had not received further medication for three days, the modeling success could be assessed by looking for signs of dryness, flushing, and scaling. Then, the mice were divided into the model control (MC) group, 0.5% LQ–CG group, 1% LQ–CG group, 2% LQ–CG group, carbomer gel (CG) group, and 0.03% tacrolimus group (Zhejiang, China) according to the randomized numerical table method, with 8 mice in each group. Starting on the 25th day of the experiment, the mice received the appropriate dose externally on the skin once a day for 14 days, depending on the grouping. The MC group did not receive any medication. Furthermore, the mice were kept in a transparent observation cage that resembled their natural habitat, and a specific amount of time was set aside after the therapy to record scratching behavior for an hour. A high-definition camera was utilized to film the mice scratching behavior for the entirety of hour-long duration. After that, these images were replayed in order to enable a thorough examination of each mice frequency of scratching.

### 2.3. Skin Histopathologic Evaluation and Histologic Analysis

Following the removal of the skin tissues, they were embedded in paraffin and fixed for 24 h in 4% paraformaldehyde. Hematoxylin-eosin (HE) was then used to stain the tissue slices. Additionally, sections were prepared for immunohistochemistry by deparaffinizing them, adding a primary antibody, and then incubating them for an additional night at 4 °C. After that, they were stained with hematoxylin and 3,30-diaminobenzidine tetrahydrochloride solution and goat anti-rabbit IgG antibody, along with affinity peroxidase reagent. The primary antibodies used for detection were Claudin-1 (AF01878) sourced from Hunan Aifang Biotechnology in China, Keratin 10 (K10, GB112105), and Aquaporin 3 (AQP3, GB11395), both of which were provided by Wuhan Servicebio Technology Co., Ltd. (Wuhan, China). To evaluate the expression of Claudin-1, K10, and AQP3 proteins in mice skin tissues, photos were obtained under a microscope.

### 2.4. Enzyme-Linked Immunosorbent Assay

Serum was separated from the mice’s blood at the end of the trial. Following the ELISA kit (Jiangsu Meimian Industrial Co., Ltd., Yancheng, China) instructions, the levels of total TNF-α, IL-1β, IL-6, IFN-γ, and IgE in serum were measured. Plotting and computation of the standard curve were carried out using the standard concentrations and matching OD values.

### 2.5. Content Measurement of Hyaluronan

After removing 50 mg of skin tissue, 500 μL of protease inhibitor-containing lysate was added, and the mixture was homogenized with an electric homogenizer. The tissue was placed in a centrifuge and spun at 12,000 rpm for 20 min at 4 °C. The supernatant was then removed and subjected to analysis in accordance with the guidelines provided by the ELISA kit (Beijing Solarbio Science and Technology Co., Ltd., Beijing, China).

### 2.6. Determination of SOD, CAT, and MDA Levels

Mice serum was obtained as described above and assayed according to the superoxide dismutase (SOD), catalase (CAT), and decreased malondialdehyde (MDA) kit manufacturer’s instructions (Nanjing Jiancheng, Nanjing, China).

### 2.7. Biocompatibility Testing

#### 2.7.1. Biosafety Studies

Body weight measurements of the mice were obtained on days 0, 3, 7, 11, and 14 following the administration of the dose, facilitating the documentation of weight changes over time. Day 14 saw the execution of every mouse, the removal of all major organs for histological analysis, and the application of biochemical assays to the mice sera. The directions provided by the kit manufacturer were followed to measure a total of four indices: alanine transferase (ALT), aspartate transferase (AST), blood urea nitrogen (BUN), and creatinine (CREA). (Nanjing Jiancheng, Nanjing, China).

#### 2.7.2. Hemolysis Test

Red blood cells were separated from mice blood via centrifugation, and hemolysis examinations were carried out in accordance with [17]. After obtaining the red blood cells, they underwent three rounds of washing in Tris buffer before being diluted to 5% (*v*/*v*). A mixture of 500 μL hydrogel and 500 μL blood erythrocytes was added to a 24-well microtiter plate. PBS served as the negative control and H_2_O as the positive control. Following mixing, the plates were statically incubated for one hour at 37 °C with 150 rpm of shaking. The microtiter plate’s contents were then centrifuged, and the 100 μL supernatant was pipetted into a 96-well plate. With the aid of an automated enzyme marker, the absorbance at 570 nm was determined. Hemolysis percentage formula: hemolysis ratio (%) = ((Ax − Ab)/(At − Ab)) × 100%, where Ax is the absorbance value of the LQ–CG experimental group, and At and Ab indicate the absorbance values of H_2_O and PBS, respectively.

### 2.8. Statistical Methods

For data analysis, GraphPad Prism 8.0 statistical software was utilized. The measurement data were presented as mean ± standard deviation (SD); for comparisons between several groups, one-way ANOVA was utilized followed by the LSD post hoc test and, for two-by-two comparisons, the Student’s *t*-test analysis was employed. A difference was deemed statistically significant at *p* < 0.05.

## 3. Results

### 3.1. Effect of LQ–CG on Skin Symptoms and Skin Water Content in Mice Subsection

**Figure 1:**
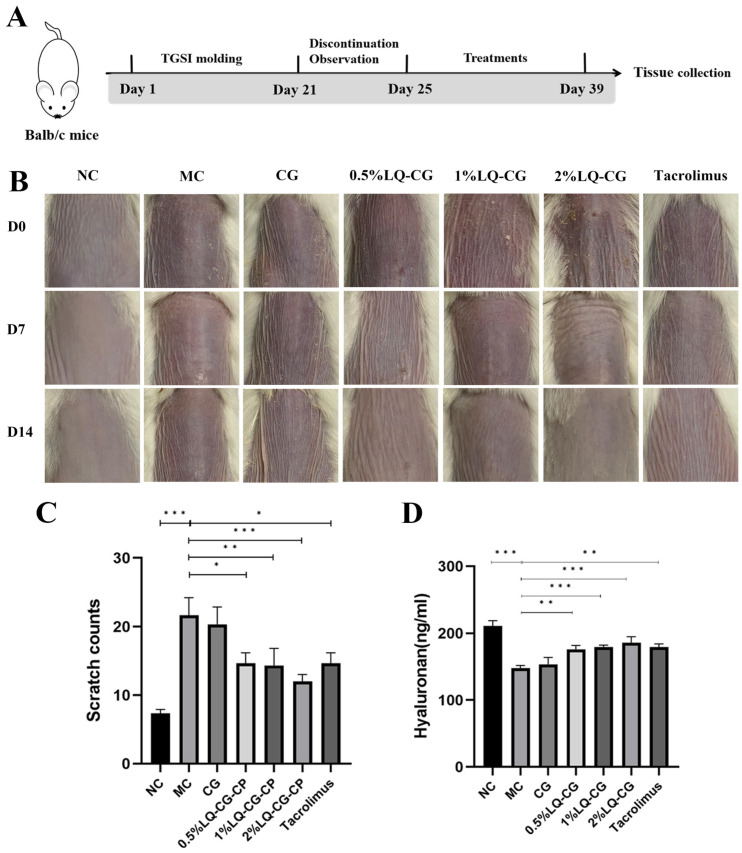
Skin changes that occur during the course of treatment. (**A**) Schematic illustration of the construction and treatment process of TGSI modeling. (**B**) Mouse skin changes during the treatment process. (**C**) Mice scratching following a 14-day treatment period. (**D**) Hyaluronan content in mouse skin tissue after 14 days of therapy. Data are presented as mean ± SD. (* *p* < 0.05, ** *p* < 0.01, *** *p* < 0.001, *n* = 6).

Figure 1A shows the construction of the TGSI model and the treatment process. Figure 1B illustrates the progression of skin alterations during treatment in each set of mice. The mice skin in the NC group is normal and untreated. The mice in the MC group had skin on their backs that showed dilated capillaries, was flushed, and covered in scattered scaly patches, as opposed to the NC group. The 0.5% LQ–CG, 1% LQ–CG, and 2% LQ–CG group skin condition of the mice improved compared to the MC group, with dryness and flushing alleviated and scaly coverage significantly disappearing. On the other hand, there was a substantial decrease (*p* < 0.05) in the itching behavior of mice in the 0.5% LQ–CG, 1% LQ–CG, 2% LQ–CG, and tacrolimus groups (Figure 1C). The mice in the 2% LQ–CG group demonstrated the highest reduction in scratches (*p* < 0.01), suggesting an especially significant advantage. Figure 1D displays the amount of hyaluronic acid in the skin tissues of the mice’s back skin during a 14-day treatment period. The figure shows that, in comparison to the MC group, the hyaluronan content of the mice’s back skin tissues increased to varied degrees in all treatment groups. The mice in the CG group had somewhat more higher levels of hyaluronan in their skin, but the hyaluronan expression in the skin of the animals in the 0.5% LQ–CG, 1% LQ–CG, 2% LQ–CG, and tacrolimus groups was significantly higher (*p* < 0.05). This implies that LQ has a significant impact on reducing dry skin.

### 3.2. Skin Histopathology and Histologic Findings

**Figure 2:**
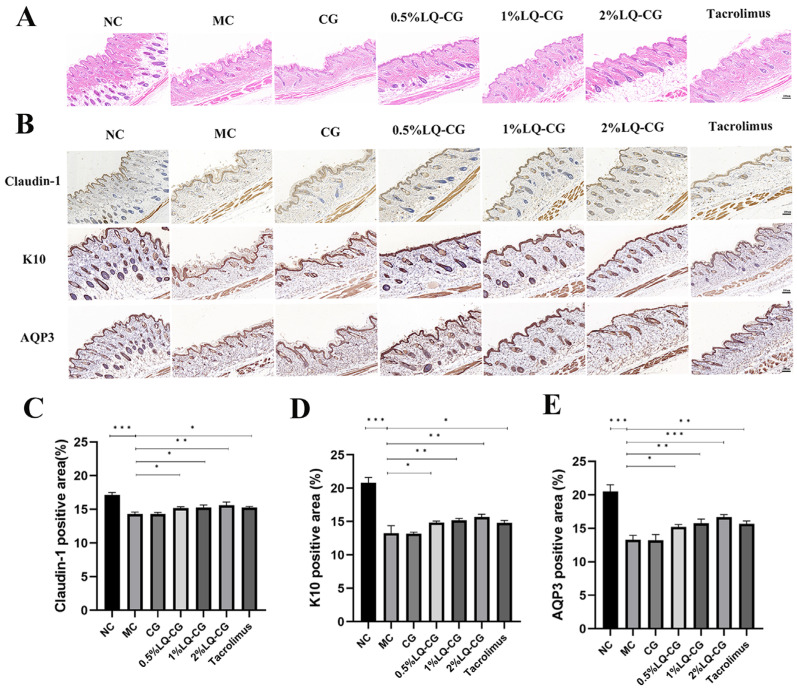
Skin slices were analyzed using immunohistochemistry and HE staining on the fourteenth day following therapy. (**A**) Photomicrographs of the skin of several treatment groups after 14 days of HE staining. (100 μm); (**B**) examination of Claudin-1, K10, and AQP3 in mouse skin tissues following a 14-day medication regimen (100 μm); (**C**) amount of Claudin-1 protein expression was measured and reported as a percentage of the entire region; (**D**) statistical evaluation of the expression of the K10 protein in mouse skin on day 14; (**E**) quantitative evaluation of the AQP3 protein in mouse skin. The data are expressed as mean ± SD (* *p* < 0.05, ** *p* < 0.01, *** *p* < 0.001, *n* = 3).

The HE results in Figure 2A demonstrate that the mice in the MC group had considerable thinning of the skin thickness and disorganized keratinization in the stratum corneum. In comparison to the MC and CG groups, the epidermal stratum corneum of the 0.5% LQ–CG, 1% LQ–CG, 2% LQ–CG, and tacrolimus showed a significant improvement, as did the degree of keratinization. Additionally, the skin thickness was restored, approaching the normal dermatopathological structure. In order to ascertain the effectiveness mechanism of LQ hydrogelation, the barrier indicators Claudin-1, K10, and AQP3 in mice skin were investigated. The proteins Claudin-1 and K10 are involved in the preservation of barrier function and tight junction stability. As demonstrated in Figure 2B–D, the LQ–CG group of mice exhibited significantly higher levels of Claudin-1 and K10 protein expression in their epidermis compared to both the MC and CG groups (*p* < 0.05). One important protein that regulates the stratum corneum’s hydration is AQP3. As demonstrated in Figure 2B,E, the LQ–CG group of mice exhibited a considerable increase in AQP3 protein expression in their epidermis as compared to the MC group (*p* < 0.05).

### 3.3. Effect of LQ–CG on Inflammatory Factors in Mice Models of TGSI

**Figure 3:**
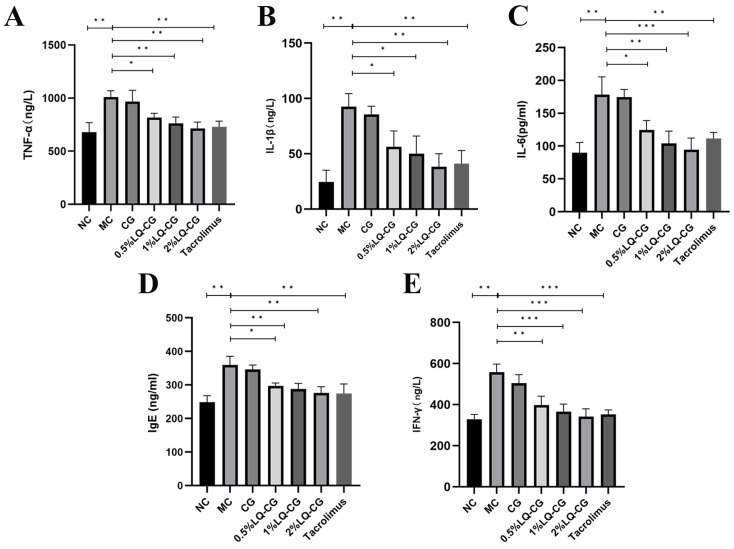
After 14 days of treatment, levels of inflammatory factors in each group of mice. (**A**) TNF-α levels in each mice group; (**B**) IL-1 β levels in each mice group; (**C**) IL-6 levels in each mice group; (**D**) IgE levels in each mice group; (**E**) IFN-γ levels in each mice group. The data are expressed as mean ± SD (* *p* < 0.05, ** *p* < 0.01, *** *p* < 0.001, *n* = 3).

By using the ELISA technique, the effects of various LQ–CG concentrations on inflammatory factors in the TGSI mice model were found. Figure 3 illustrates that the MC group had significantly greater serum levels of TNF-α, IL-1β, IL-6, IgE, and IFN-γ in comparison to the blank group (*p* < 0.05). TNF-α, IL-1β, IL-6, IgE, and IFN-γ levels were not significantly decreased after 14 days of administration in the carbomer gel group when compared to the model group. However, there was a dose-dependent significant reduction in TNF-α, IL-1β, IL-6, IgE, and IFN-γ levels in the serum of the 0.5% LQ–CG, 1% LQ–CG, and 2% LQ–CG groups (*p* < 0.05).

### 3.4. Effect of LQ–CG on Serum SOD, CAT, and MDA Levels in TGSI Mice

**Figure 4:**
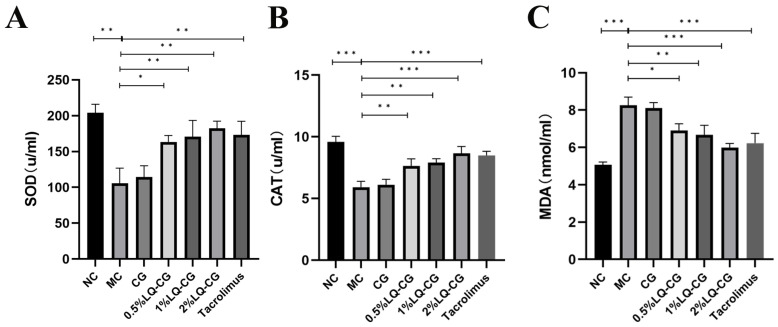
The number of components linked to oxidative stress in each group of mice were measured after 14 days of treatment. (**A**) Levels of SOD in each group of mice; (**B**) level of CAT in each group of mice; (**C**) levels of MDA in each group of mice. Data are presented as mean ± SD (* *p* < 0.05, ** *p* < 0.01, *** *p* < 0.001, *n* = 3).

Using appropriate kits, the amount of oxidative damage found in the serum of the mice was determined. Figure 4 illustrates that the mice in the MC group had considerably lower levels of SOD and CAT activities in their serum compared to the NC group, but their MDA content was significantly increased (*p* < 0.05), while the serum activities of SOD and CAT were significantly increased and the MDA content was significantly decreased in the mice in the 0.5% LQ–CG, 1% LQ–CG, 2% LQ–CG, and tacrolimus groups (*p* < 0.05). No significant changes were observed in the levels of SOD, CAT, and MDA in the CG group compared to the MC group (*p* > 0.05).

### 3.5. Biosafety Studies

**Figure 5:**
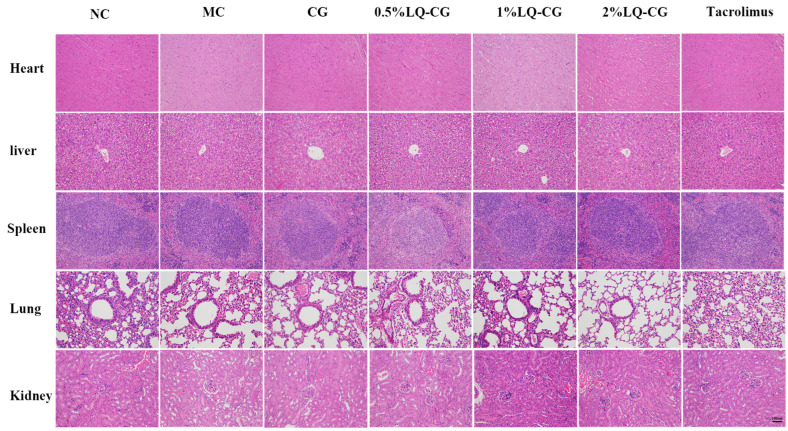
Day 14 following various treatments, HE staining of the heart, liver, spleen, lung, and kidney (magnifications, ×100).

To confirm the safety of LQ–CG, the in vivo toxicity in treated mice was additionally monitored. Vital organs, including the liver, heart, spleen, lungs, and kidneys, were stained with HE double staining on day 14 after treatment in order to evaluate the pathological alterations in the visceral tissue. When compared to the NC group, there were no appreciable histopathologic alterations (Figure 5). The findings imply that LQ–CG has no harmful or damaging impact on the organs.

### 3.6. In Vivo Toxicity and In Vitro Blood Compatibility of LQ–CG

**Figure 6:**
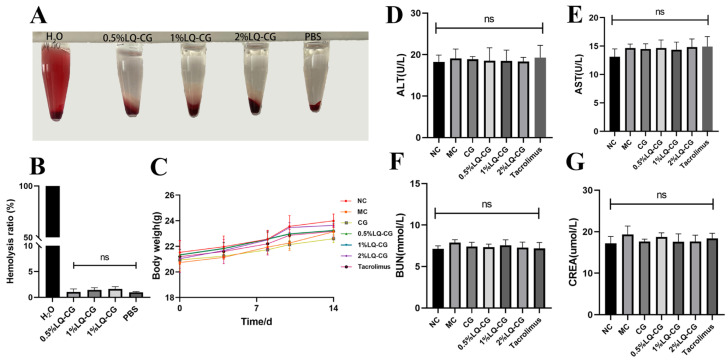
Testing for biosafety. (**A**) Hemolysis test photos; (**B**) hemolysis ratio quantitative data is shown as mean ± standard deviation; (**C**) after the mice have received therapy for 14 days, they are weighed; (**D**–**G**) at the end of treatment, blood biochemical indices (ALT, AST, BUN and CRE) for mice in each group. Data are presented as mean ± SD. “ns” denotes no discernible change. (*p* > 0.05, *n* = 3).

Following a 14-day treatment period, no statistically significant variation was observed in the mice’s body weight between the treated and NC groups (*p* > 0.05). This suggests that LQ–CG did not have any discernible impact on overall health (Figure 6C). Furthermore, the safety of LQ–CG was assessed using blood biochemical parameters. The findings indicated that there was no statistically significant difference in the changes in biomarkers in the kidneys and liver with LQ–CG, including ALT, AST, BUN, and CREA (*p* > 0.05) (Figure 6D–G). The safety of LQ–CG for internal organs is further supported by this study. In addition, an experiment was conducted where erythrocytes were combined with LQ–CG and incubated for one hour. The findings revealed that, whereas the positive control of H_2_O was brilliant red, there was no discernible hemolysis in the LQ–CG group or the negative control group (Figure 6A). The hemolysis ratio of the various LQ–CG concentrations (0.5%, 1%, and 2%) were 0.52%, 1.18%, and 1.77%, respectively, according to data analysis, and these values were below the hemolysis level of 5.0% (Figure 6B). This suggests that LQ–CG has outstanding compatibility with blood.

## 4. Discussion

The purpose of this study was to examine the effectiveness and potential therapeutic mechanism of LQ–CG in TGSI mice. The findings demonstrated that LQ–CG was highly effective in reducing the symptoms, repairing the skin barrier, and lowering the oxidative stress level in TGSI mice.

The proliferation and differentiation of keratinocytes are impacted by prolonged or uncontrolled topical GC treatment, which inhibits collagen synthesis and thins the epidermis [18]. Simultaneously, it will result in aberrant glucose and protein metabolism linked to the skin, which will impact skin growth and repair. Additionally, it will lead the skin to lose its natural barrier function, leaving the skin more vulnerable to infections and outside stimuli [6,19]. Patients with TGSI often experience recurrent episodes that have a significant negative impact on their physical and emotional well-being. According to a systematic review of dermatology patients, between 21% and 84% were afraid to apply topical steroids [20]. Psychological stress disrupts the skin barrier equilibrium and encourages the emergence and progression of skin inflammation, so starting a vicious cycle [21,22]. As such, it is critical to treat TGSI as soon as possible or to actively prevent development.

In order to examine how LQ–CG affected TGSI, successful models of the disease were constructed in mice. Three days after the mice modeling medicine ended, their skin looked flushed, dry, and covered with little scales. Following a 14-day duration of LQ–CG application, the mice in the MC group continued to exhibit flushing, dryness, and sporadic scaly covering on their skin, but the LQ–CG and tacrolimus groups showed a notable improvement in skin appearance. Skin problems, including dry, flaky, or sensitive skin, may arise from dysregulation of the skin barrier function, which can also set off an inflammatory cascade [23]. Furthermore, skin inflammation, which can be both a cause and a result of dry skin, frequently sets off a vicious cycle of additional barrier disruption and can produce pruritus [24]. In order to further confirm the therapeutic advantages of LQ–CG, behavioral analysis was also included in this investigation by observing the mice scratching behavior. Itching is a common complaint among patients with TGSI, which has a detrimental impact on quality of life [25]. Consequently, a statistical examination of the mice’s scratching activity revealed that the number of scratches made by the animals in the CG group was not significantly different from that of the mice in the MC group. The number of scratches in the groups receiving tacrolimus, 0.5%, 1%, and 2% LQ–CG was considerably lower than that of the MC group, indicating the effectiveness of LQ–CG in reducing itching symptoms. This not only provides compelling evidence for the antipruritic impact of LQ–CG, but also shows how valuable it may be in improving patients’ quality of life.

The epidermis, dermis, and subcutaneous tissue make up the skin. The functions of the skin as a whole are influenced by the distinct roles that each layer performs in pre-serving the skin’s integrity. Adipocytes and connective tissue make up the majority of the subcutaneous tissue [26]. The presence of adipocytes can affect the general health of the skin by offering protection and storing energy [27]. When examining and analyzing tissue structure, HE staining is a more practical and intuitive method. Therefore, the mice’s skin was examined pathologically. Under a microscope, examination of the HE staining content revealed that the mice in the MC group had noticeably thinner skin tissues overall, with fewer subcutaneous lipids, and the surface stratum corneum’s keratinization was aberrant and disorganized. On the other hand, the disordered skin tissues in the tacrolimus and LQ–CG groups showed a considerable improvement, and the stratum corneum’s organization and skin thickness tended to be normal and orderly, suggesting that the skin’s barrier function was being efficiently repaired.

The skin, the biggest organ in the human body, serves as an internal barrier to keep the organism safe. It can successfully protect against microbiological and environmental threats, but it can also preserve internal equilibrium to support general health [28]. There is currently no consensus regarding the pathogenesis of TGSI; however, damage to the skin barrier is thought to be a contributing factor [19]. The stratum corneum of the epidermis and its tight connections make up the skin barrier. The stratum corneum is the outermost layer of the skin. It is made up of keratinocytes and various natural moisturizing agents that work together to create a “brick wall structure”, which acts as a mechanical barrier and a powerful defense against the entry of pathogenic microorganisms from the outside [29]. Furthermore, its structure prevents the epidermis from losing moisture by retaining it. Tight junctions are composed of Claudins, Occludin, and some cytoplasmic scaffolding proteins, such as the Occludin ribbon proteins Z0-1 and Z0-2 [30]. The epidermal and subepidermal layers of the skin contain tight junctions that serve to close intercellular junction complexes between nearby keratinocytes in the granular layer. This process preserves the tight junctions between skin cells and, consequently, the integrity of the skin barrier. Mice skin was stained immunohistochemically in order to gain a better understanding of the potential mechanism underlying the effectiveness of LQ–CG. Positive outcomes demonstrated that K10 and Claudin-1 protein expression was considerably higher in the LQ–CG group compared to the MC and CG groups. The structural proteins known as keratins serve as cytoskeletons and platforms for cell signaling within the interior of keratin forming cells. K10 stands out among them all as it is frequently utilized as a marker of proper cell differentiation and has been linked to both inflammatory response and skin barrier function at the expression level [31]. On top of that, Claudin-1 is essential for the preservation of the mammalian epidermal barrier. Claudin-1 deficient mice exhibited severe problems in the skin barrier and died within a day after birth from dehydration and aberrant skin differentiation, according to research by Furuse et al. on Claudin-1 deficient mice models [32]. Rosac et al. have shown that Claudin-1 expression down-regulation is a major contributor to the development of skin inflammation and can result in anomalies in intercellular tight junctions [33]. In addition, a dose-dependent association was found between Claudin-1 expression and the degree of skin inflammation, as well as a favorable correlation with skin barrier function [34]. According to the findings, LQ–CG may help restore skin barrier function by boosting the expression of the proteins K10 and Claudin-1.

Aquaporins are a family of transporter proteins in cell membranes, and the main Aquaporins in the skin are AQP3 [35]. AQP3 plays a key role in the maintenance of skin homeostasis [36]. To be more precise, AQP3 functions as a water channel protein that makes it easier for water molecules to move across cell membranes, assisting cells in maintaining water balance and intercellular signaling. Moreover, AQP3 plays a role in controlling the metabolic processes of skin cells and encouraging the development and maintenance of keratinocytes. Research has demonstrated that variations in AQP3 expression impact the function of the skin barrier, resulting in the emergence of inflammatory skin conditions [37]. AQP3 plays a key role in repairing the skin barrier in this pathway. According to Hara et al., mice lacking AQP3 had reduced skin elasticity and epidermal cell water transport ability, which postponed barrier restoration and wound healing [38]. Mice lacking the 24 dehydrocholesterol reductase gene displayed water retention in their epidermis due to increased production of AQP3 in their skin, resulting in firm, wrinkle-free skin, as demonstrated by Mirza et al. [39]. In the TGSI mice model, it was observed that LQ–CG markedly increased the expression of AQP3 in the epidermis of the mice. Overall, LQ treatment restored the down-regulation of AQP3 caused by GC. This implies that LQ–CG has a major efficacious effect on the cutaneous symptoms of mice with TGSI. Furthermore, the hyaluronan content of the skin is crucial for maintaining the keratin forming cells’ flexibility and water-binding ability as well as the structural elasticity and water binding properties of the dermal layer and epidermis [40]. The LQ–CG group increased the content of skin hyaluronan to varying degrees in comparison to the MC and CG groups, based on the results of the skin hyaluronan assay in mice. The 2% LQ–CG group showed a greater rise in skin hyaluronan content than the tacrolimus group. As anticipated, LQ may boost the expression of the AQP3 protein and increase the amount of hyaluronan in the skin, strengthening the skin barrier.

In TGSI, skin injury results in the production of inflammatory factors by cells, exacerbating the onset of skin inflammation. While TNF-α, IL-1β, and IL-6 are significant pro-inflammatory cytokines, IgE and IFN-γ have an impact on immune cell contacts, immunoregulatory processes, and the regulation and intensification of the inflammatory response [41]. According to the available data, LQ–CG treatment substantially eased the inflammatory response in TGSI by dramatically reducing the expression of TNF-α, IL-1β, IL-6, IgE, and IFN-γ. This implies that, in TGSI mice, LQ plays a part in regulating the skin’s immune response and reducing the inflammatory response.

Topical GC disrupts the function of the epidermal barrier by causing oxidative stress [42]. According to Ulla et al., dexamethasone caused the generation of reactive oxygen species and markedly reduced the expression of CAT and SOD in mice myocytes [43]. In a similar vein, intracellular reactive oxygen species buildup, down-regulation of mitochondrial membrane potential, and decreased cysteine asparaginase activity were seen in MC3T3-E3 cell culture upon the addition of dexamethasone [44]. Antioxidant enzymes work in concert to alter the homeostatic state of oxidative stress, which makes their synergistic effect crucial. This investigation found that serum expression levels of SOD and CAT were significantly lower in the MC group, whereas malondialdehyde expression was significantly higher. Nevertheless, the treatment of LQ–CG greatly alleviated this negative scenario. The enhanced activity of LQ–CG on the antioxidant defense system thus represents a further potential mechanism that could aid in the restoration of TGSI skin barrier function.

Throughout the study, the mice’s daily body weights in each group stayed similar. HE staining experiments were conducted to examine major organs, and no signs of toxic reactions or severe organ damage were observed. Furthermore, the regular blood indices showed that ALT, AST, BUN, and CREA were all within normal ranges. In the meantime, the hemolysis test findings, the LQ–CG hemolysis ratio, was within the normal range, suggesting that the drug had little hemolytic effects on mice and was generally safe.

## 5. Conclusions

In summary, LQ–CG demonstrated encouraging outcomes in the management of TGSI, which were attributed to its function in restoring the skin barrier in addition to its suppression of inflammatory factors and reduction of oxidative stress levels. Therefore, these results highlight the possibility that LQ–CG could represent a novel approach to TGSI treatment as well as a viable choice for upcoming clinical uses. Nevertheless, the pathophysiology of TGSI remains unclear, and comprehensive cellular molecular studies have not yet been carried out. It is advised that further research be performed in the future on the pathophysiology of the illness.

## Data Availability

The data that support the findings of this study are available from the corresponding author upon reasonable request.

## Supplementary Materials

The following supporting information can be downloaded at: https://www.mdpi.com/article/10.3390/pharmaceutics16081001/s1. Figure S1. The liquiritin structural formula Molecular formula: C21H22O9 Molecular weight: 418.39.
