# Supplementary material for: Therapeutic Effect of Liquiritin Carbomer Gel on Topical Glucocorticoid-Induced Skin Inflammation in Mice
Source: Pharmaceutics. 2024 Jul 28;16(8):1001. doi: 10.3390/pharmaceutics16081001 (PMC11359290; doi:10.3390/pharmaceutics16081001)
Supplement: Supplementary file 1 [file pharmaceutics-16-01001-s001.zip › pharmaceutics-3117261-supplementary.pdf]

1.The liquiritin structural formula

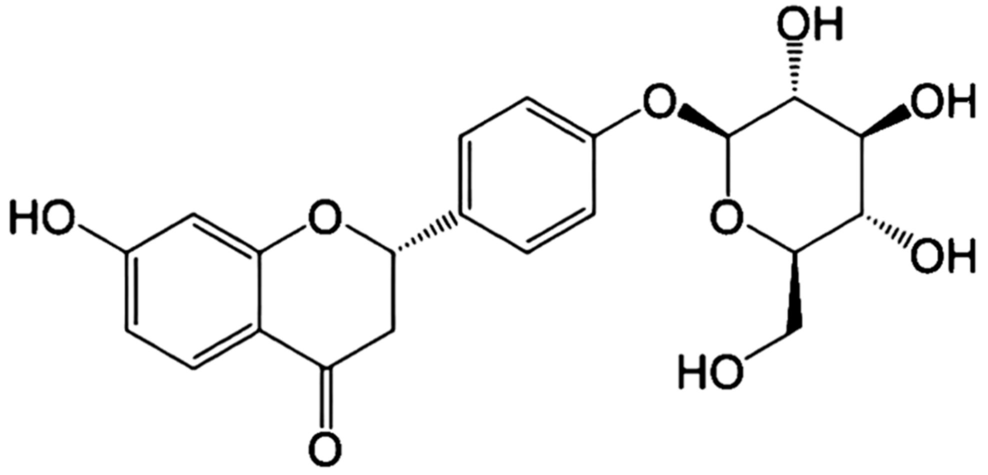

Figure S1. The liquiritin structural formula

Molecular formula: $C_{21}H_{22}O_9$ Molecular weight: 418.39
